# Secondary Immune Thrombocytopenia Due to Mycoplasma pneumoniae Without Clinically Significant Evidence of Active Infection

**DOI:** 10.7759/cureus.23551

**Published:** 2022-03-27

**Authors:** Changsu L Park, Bryan Margaria, Muhammad Husnain

**Affiliations:** 1 Department of Medicine, MetroHealth Medical Center, Cleveland, USA

**Keywords:** thrombocytopenia, infection, mycoplasma, secondary itp workup, itp

## Abstract

Immune thrombocytopenia (ITP) is a leading cause of isolated thrombocytopenia characterized by autoantibody-mediated destruction of platelets, impaired megakaryocyte function, and pathologic T-cell recognition of platelet antigens. Several triggers for ITP have been identified. Treatment of the inciting cause decreases the antibodies responsible for molecular mimicry, and these cases are usually associated with a better outcome with a decreased probability of progression to chronic ITP. *Mycoplasma pneumoniae* infection is known to have extrapulmonary manifestations, and growing evidence suggests it can be a cause of secondary ITP. Many of the described cases report evidence of a pulmonary infection with severe mucosal bleeding. Here, we describe an interesting case of a patient presenting with isolated thrombocytopenia with mild mucosal bleeding, later found to be positive for *Mycoplasma *immunoglobulin M without clinically significant evidence of active infection. Currently, mycoplasma testing is not routinely performed as a workup for ITP. However, clinicians may consider this before proceeding with more aggressive treatment for refractory ITP (i.e., prolonged immunosuppression, splenectomy). This case illustrates that mild/asymptomatic *Mycoplasma* infection can also be associated with ITP.

## Introduction

Immune thrombocytopenia (ITP; previously known as idiopathic thrombocytopenic purpura) is a leading cause of isolated thrombocytopenia with a prevalence of 9.5 per 100,000 in adults [1]. The disease process is characterized by autoantibody-mediated destruction of platelets, impaired megakaryocyte function, and pathologic T-cell recognition of platelet antigens [1]. ITP is subcategorized into three phases: newly diagnosed (<3 months), persistent (3-12 months), and chronic (>12 months) disease, each with respective treatment strategies and anticipated complications [2]. Bleeding-related events are most common in newly diagnosed ITP (2.67 per patient-year) and lowest in chronic ITP (0.73 per patient-year), with an overall rate of 1.08 per patient-year across the board [3]. The rate of intracerebral hemorrhage (ICH) and severe bleeding in adults with ITP have been reported to be 1.4% and 9.6%, respectively [4]. However, the rate and severity of bleeding diatheses in these studies were highly variable and did not always correlate with the degree of thrombocytopenia. Intriguingly, patients with ITP also had a higher risk for thromboembolic events, hematologic malignancy, and 1.5-fold increased mortality compared to the general population [5,6].

Primary ITP accounts for 80% of all cases and is usually an acquired autoimmune disorder against common platelet glycoproteins such as GPIIb/IIIa. Secondary ITP is associated with other underlying diseases including autoimmune (systemic lupus erythematosus, rheumatoid arthritis), infectious (hepatitis C, human immunodeficiency virus, *Helicobacter pylori*, cytomegalovirus, varicella, upper respiratory tract infections), malignant disorders (chronic lymphocytic leukemia), and medications, which produce antibodies that cross-react with platelet antigens [7]. In several instances, treatment of the inciting cause has been shown to improve the clinical course, likely by decreasing the antibodies responsible for this molecular mimicry and thereby decreasing the probability of progression to chronic ITP [1]. Therefore, further understanding of the risk factors for secondary ITP will allow clinicians to better recognize, treat, and anticipate the disease course.

*Mycoplasma pneumoniae* is the most common cause of atypical pneumonia and has been estimated to account for up to 25% of all community-acquired pneumonia [8]. However, only 5-10% of patients infected with *Mycoplasma* develop pneumonia and most cases are subclinical or mild and do not require clinical attention [9]. Extrapulmonary manifestations of *Mycoplasma* have been extensively described in the literature, including hematologic disorders such as autoimmune hemolytic anemia, thrombotic thrombocytopenic purpura, disseminated intravascular coagulation, and hemophagocytic syndrome [10]. While *Mycoplasma* infection has been associated with pathology in all organs irrespective of bacterial infiltration, the exact mechanism for its diverse clinical presentation is largely unknown. Several case reports suggest that *Mycoplasma* infection may be associated with ITP [11-14]. In the limited cases described in the literature, patients presented with clinically significant respiratory tract infection and were found to have ITP often associated with severe hemorrhagic complications and a refractory clinical course.

Here, we describe an interesting case of a patient presenting with isolated thrombocytopenia with mild mucosal bleeding, later found to be positive for *Mycoplasma* immunoglobulin M (IgM) without clinically significant evidence of active infection.

## Case presentation

A 54-year-old Caucasian woman with a medical history of atrial fibrillation (on warfarin), hypertension, type 2 diabetes mellitus, obstructive sleep apnea, seasonal allergies, recurrent cellulitis of the lower extremity (on suppressive amoxicillin therapy), obesity (140 kg, 50 kg/m^2^), and heart failure with preserved ejection fraction presented to an emergency department in an urban academic center with the chief complaint of intermittent pleuritic chest pain and fatigue for one day. A review of the systems was positive for rhinorrhea, sore throat, ear fullness (consistent with her seasonal allergies), four-day history of dry cough, and chronic shortness of breath. Intriguingly, she noted increased bleeding from mucosal surfaces such as the nose and gums for one month. She also endorsed chills, malaise, blood in stool, diarrhea, nausea, vomiting, myalgias, joint pain, tingling in the extremities, and headaches. There was no personal or family history of bleeding disorders. She had a remote 10 pack-year smoking history, negative for alcohol or substance abuse. Vital signs were within normal limits breathing room air. Physical examination was significant for tender splenomegaly but negative for bruising, friable mucosa, edema, rales, or rhonchi. Laboratory findings were significant for isolated thrombocytopenia of 28,000/µL (prior platelet counts were all above 100,000/µL; one month prior 159,000/µL). Chest X-ray did not reveal consolidation or pulmonary edema. The patient was admitted for further workup of thrombocytopenia.

Her platelets reached a nadir of 26,000/µL on days two and three of admission. An extensive thrombocytopenia workup including anti-nuclear antibody, Epstein-Barr virus, human immunodeficiency virus, hepatitis C, and thyroid-stimulating hormone was unrevealing. Hemolysis and thrombotic microangiopathy workup were also normal, including Coombs test, fibrinogen, lactate dehydrogenase, total bilirubin, and D-dimer. A peripheral smear showed thrombocytopenia without platelet clumping but was otherwise normal. Complete blood count showed 6,000/µL white blood cells, 12.2 g/dL hemoglobin (reference range: 12.0-15 g/dL), and a mean corpuscular volume of 88 fL (reference range: 80-100 fL). Computed tomography (CT) of the abdomen/pelvis was negative for lymphadenopathy, but mild splenomegaly of 14.2 cm was noted. Flow cytometry of peripheral blood was negative for clonal proliferation. Nutritional studies for thrombocytopenia showed normal iron/ferritin and vitamin B12 level of 225 pg/mL (normal: >300 pg/mL, borderline: 200-300 pg/mL, deficient: <200 pg/mL).

At this point, ITP was high on the differential diagnosis as other common etiologies of thrombocytopenia such as lupus, viral infection, and malignancies had been ruled out. Intriguingly, the patient’s mean platelet volume (MPV) was 10.9 fL (reference range: 7.5-11.2 fL), increasing from 8.9 fL one month prior. *H. pylori* stool antigen was negative. The patient was found to have a positive *Mycoplasma* IgM with a titer level of 902 (normal: <770). A positive value indicates recent acute infection with *Mycoplasma*. The patient was started on a two-week prednisone taper (starting at 40 mg daily; a lower dose than the conventional 1 mg/kg/day given her frequent history of decompensated heart failure) and doxycycline for seven days. After one month, her platelet had increased to 148,000/µL with an MPV of 9 fL. Her platelet counts remained >150,000/µL over a one-year follow-up.

## Discussion

ITP is a disease that can be caused by primary sensitization to glycoproteins IIb/IIIa and Ib/IX or secondary etiologies, including medication, infectious, malignant, and other autoimmune pathologies. Common infectious triggers for the disease that are frequently referenced in existing literature include *H. pylori*, cytomegalovirus, and varicella-zoster virus. This case illustrates that ITP could also be caused by a less commonly described pathogen, *Mycoplasma*, even in the absence of traditional symptoms such as lower respiratory tract infection or fever.

A few confounding factors remain in the retrospective clinical analysis of this case. Our patient was on chronic suppressive amoxicillin, which is also associated with thrombocytopenia via the hapten-dependent antibody process. Drug-induced thrombocytopenia (DIT) is difficult to distinguish from ITP and some guidelines recommend treating severe cases with steroids in the initial stages of workup. However, the treatment of DIT is usually cessation of the causative agent rather than corticosteroids [15]. A retrospective analysis of 309 patients with well-documented DIT showed that corticosteroids did not affect the clinical course [16]. Furthermore, corticosteroids, plasma exchange, and intravenous immunoglobulin, which are studied treatments for ITP were not superior for vancomycin-induced thrombocytopenia, the pathophysiology of which is also through hapten formation [17]. Given that our patient responded promptly to corticosteroids and amoxicillin was not discontinued during this period, DIT and ITP secondary to amoxicillin remain lower on the differential diagnosis.

Another cause of thrombocytopenia is vitamin B12 deficiency. While further studies are required to apply in routine clinical practice, MPV may be a useful tool in differentiating the different causes of thrombocytopenia. A low MPV is a marker of B12 deficiency, and repletion of B12 after one month significantly increases MPV [18,19]. Conversely, an MPV of >8.8 fL is associated with thrombocytopenia due to destructive causes including ITP. This study showed that the sensitivity and positive predictive value at this cutoff were both 89% [20]. High MPV in our patient at presentation (10.9 fL) and subsequent decrease after steroids (9 fL) may indicate that ITP is the culprit pathology rather than vitamin B12 deficiency. Furthermore, our patient did not have other more common signs of vitamin B12 deficiency such as macrocytic anemia, and the clinical response to steroids as measured by platelet count was more consistent with the time course of ITP.

## Conclusions

There are several reports of *Mycoplasma* having an association with ITP, as discussed previously. Interestingly all cases described clinically active *Mycoplasma* as a part of the presentation. This case presented as isolated thrombocytopenia with chest pain, headaches, myalgias, but no radiographical or clinical signs of infection. Further studies are required to determine whether treating the *Mycoplasma* infection with or without concomitant corticosteroids alters the clinical course. Currently, *Mycoplasma* testing is not routinely performed as a workup of ITP. However, clinicians may consider this before proceeding with more aggressive treatment for refractory ITP (i.e., prolonged immunosuppression and splenectomy) as this case illustrates that mild/asymptomatic *Mycoplasma* infection can also be associated with ITP.
